# Amino-substituted diazocines as pincer-type photochromic switches

**DOI:** 10.3762/bjoc.9.1

**Published:** 2013-01-02

**Authors:** Hanno Sell, Christian Näther, Rainer Herges

**Affiliations:** 1 Otto-Diels Institut für Organische Chemie, Christian-Albrechts-Universität zu Kiel, Otto-Hahn-Platz 4, 24418 Kiel, Germany; 2 Institut für Anorganische Chemie, Christian-Albrechts-Universität zu Kiel, Max-Eyth-Str. 2, 24418 Kiel, Germany

**Keywords:** azobenzene, diazocine, molecular pincer, molecular switches, photochromic compound

## Abstract

Azobenzenes are robust, reliable, and easy to synthesize photochromic switches. However, their high conformational flexibility is a disadvantage in machine-like applications. The almost free rotation of the phenyl groups can be restricted by bridging two *ortho* positions with a CH_2_CH_2_ group, as realized in the dihydrodibenzo diazocine framework. We present the synthesis and properties of 3,3’-amino- and 3,3’-acetamido substituted diazocines. Upon irradiation with light of 405 and 530 nm they isomerize from the *cis* to the *trans* configuration and back, and thereby perform a pincer-like motion. In the thermodynamically more stable *cis* isomer the lone pairs of the amino nitrogen atoms point towards each other, and in the *trans* form they point in opposite directions. The distance between the amino nitrogen atoms changes between 8 Å (*cis*) and 11 Å (*trans* isomer).

## Introduction

**Figure 1:**
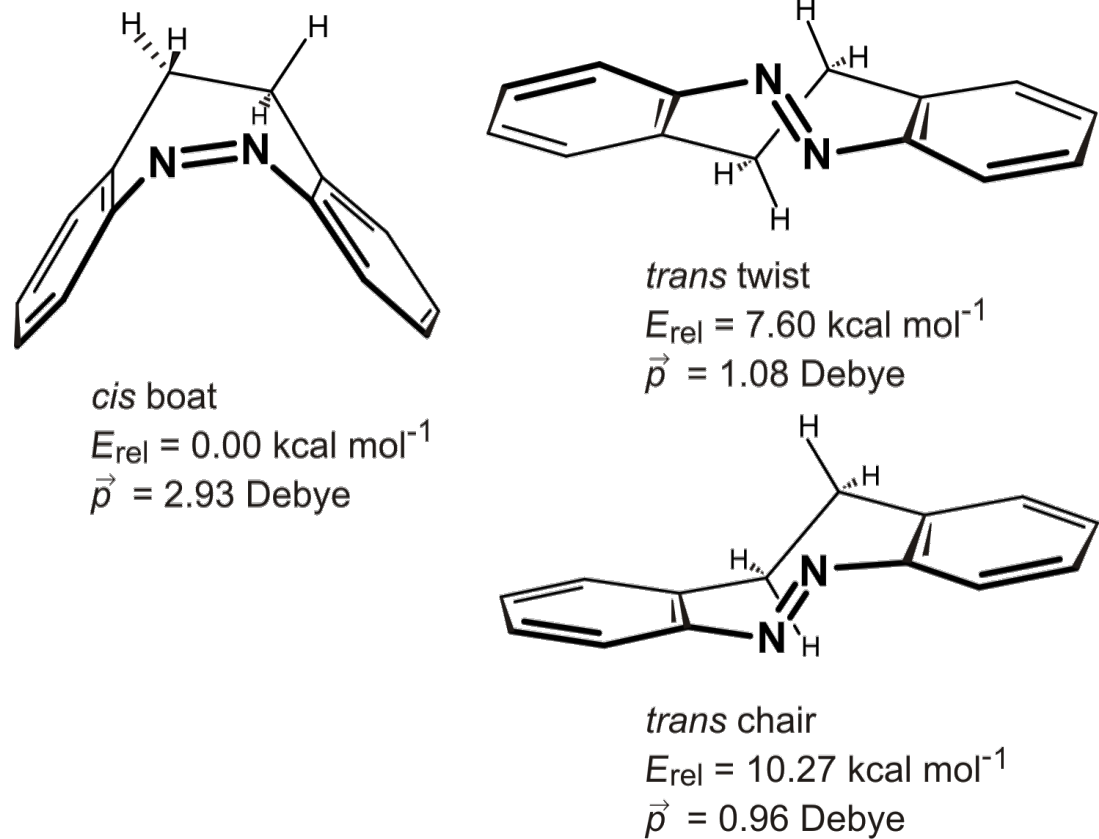
Configurations and conformations of 5,6-dihydrodibenzo[*c*,*g*][1,2]diazocine (**1**), and DFT (B3LYP/6-31G*) calculated energies (kcal mol^−1^) relative to the most stable *cis* boat form. Dipol moments are given in Debye.

Azobenzenes probably are the most frequently used photochromic switches in chemistry. They are employed as molecular actuators to drive a number of dynamic machine-like functions [1]. To achieve sophisticated engineering tasks such as directed motion at the molecular level [2–3], the geometry change and the force induced during *cis*–*trans* isomerization has to be coupled to the environment. In the macroscopic world, therefore, machines are made from stiff materials. Azobenzene, however, is a rather floppy molecule. Both phenyl rings can rotate with a low activation barrier, and isomerization of the *trans* form can occur in two different directions, forming two different isomers (enantiomers in the parent system) [4]. Power transmission to neighbouring molecules is inefficient because of energy transfer to internal conformational motion. The first and probably most simple measure to make azobenzene stiffer would be to prevent the phenyl groups from rotating. Connecting both rings with each other via an alkane bridge is probably the most straightforward way to achieve that. Such a molecule, 5,6-dihydrodibenzo[*c*,*g*][1,2]diazocine (**1**), has been known for more than a hundred years [5]. However, only recently we discovered that the photophysical properties of **1** (quantum yields, photostationary states) are superior to those of parent azobenzene, and most of its derivatives [6–8]. In contrast to azobenzene, diazocine **1** is most stable in its *cis* configuration, which has a boat conformation. The *trans* isomer comes in two conformations: a chair and a twist form, of which the twist is more stable (Figure 1).

Proper substitution of the diazocine molecular framework is necessary to control the interaction with the environment or with other molecules. Therefore, we explore different approaches to prepare diazocine derivatives. Since the nomenclature is not unambiguous and, hence, potentially confusing, we refer to 5,6-dihydrodibenzo[*c*,*g*][1,2]diazocine derivatives as 2,2’-ethylene-bridged azobenzenes (EBABs).

## Results and Discussion

### Synthesis

**Scheme 1:**
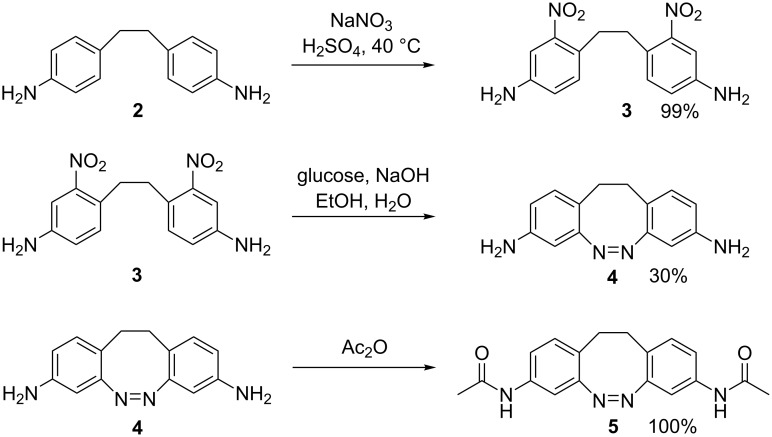
Synthesis of 3,3’-diamino-EBAB **4** and its acetamide derivative **5**.

Woolley et al. very recently published the synthesis of a 4,4’-diamino-2,2’-ethylene-bridged azobenzene (4,4’-diamino-EBAB), which exhibits excellent photophysical properties [9]. In planning our synthesis (and not yet being aware of the results of the above authors) we were concerned about the fact that amino substituents in the 4-position with respect to the azo group would impair photochemical conversion, as this is known for 4-amino-substituted azobenzenes [10]. We therefore set out to synthesize 3,3’-diamino-EBAB **4** and derivatives thereof. Key steps of the synthesis are the introduction of the substituents and formation of the azo bond. Several approaches were evaluated, changing the order of the steps and the groups from which the azo unit was generated. The preferred procedure starts from commercially available 1,2-bis(4-aminophenyl)ethane (**2**). Nitration proceeds almost quantitatively in *ortho* position to the ethylene bridge, forming 1,2-bis(2-nitro-4-aminophenyl)ethane (**3**) [11]. Intramolecular reductive coupling of the nitro groups to form the azo unit proceeds with notoriously low yields. The most frequently used procedure using Zn as the reducing agent in Ba(OH)_2_ [12] or NaOH [13] gives irreproducible and low yields varying from 2% to not more than 19%. Glucose, however, in basic ethanolic solution turned out to furnish the azo compound **4** reproducibly in more than 20% yield. The acetamide derivative **5** is formed by treatment of 3,3’-EBAB **4** with acetic anhydride (Scheme 1).

**Figure 2:**
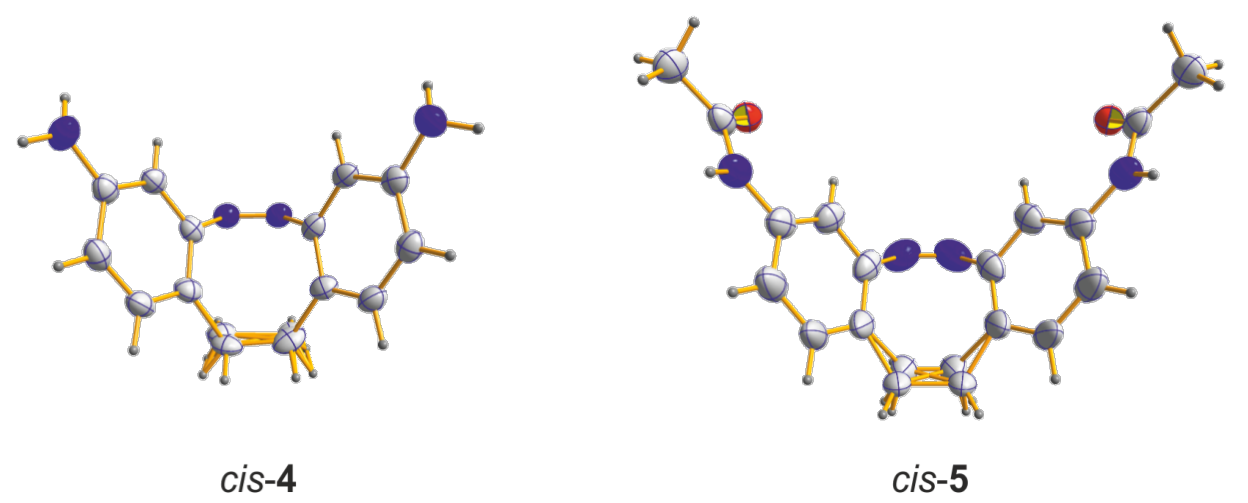
Crystal structures of the *cis* isomers of 3,3’-diamino-EBAB **4** and its acetamide derivative **5**. The atoms of the ethylene bridge are disordered.

The structures of the products were confirmed by ^1^H and ^13^C NMR spectroscopy, as well as X-ray crystallography (Figure 2). As in the parent system **1** the amino and acetamido derivates **4** and **5** are thermodynamically most stable in their *cis* form.

### Photochromic Properties

*trans*-Azobenzene exhibits a high-intensity π–π* band at λ_max_ = 316 nm (ε = 22,000 L∙M^−1^∙cm^−1^) and a symmetry-forbidden n–π* band at 444 nm with a very low extinction coefficient (ε = 440 L∙M^−1^∙cm^−1^) [10]. Irradiation with UV-light of 365 nm converts the *trans* to the *cis* isomer. The n–π* absorption of the nonplanar *cis*-azobenzene is not formally symmetry forbidden. Even though it has a rather low extinction coefficient (1250 L∙M^−1^∙cm^−1^), irradiation into the n–π* band leads to complete conversion back to the *trans* isomer [14]. Photoswitching of 5,6-dihydrodibenzo[*c*,*g*][1,2]diazocine (unsubstituted EBAB **1**), however, is performed differently [6]. Conversion of *cis* to *trans*, as well as isomerization of *trans* to *cis*, is achieved by irradiation into the corresponding n–π* bands. This is possible because the n–π* bands appear well separated at different wavelengths (*cis*: 404 nm, *trans*: 490 nm) and the transitions are allowed (albeit weak) in both isomers. Since substituents in the *meta* position are known to interact less efficiently with each other than those in the *ortho* or *para* positions we decided to examine EBABs that are 3,3’-substituted, hoping that the excellent switching properties of the parent system would be retained. For determination of the ratio of isomers in photostationary states we used ^1^H NMR (for details see Supporting Information File 1).

**Figure 3:**
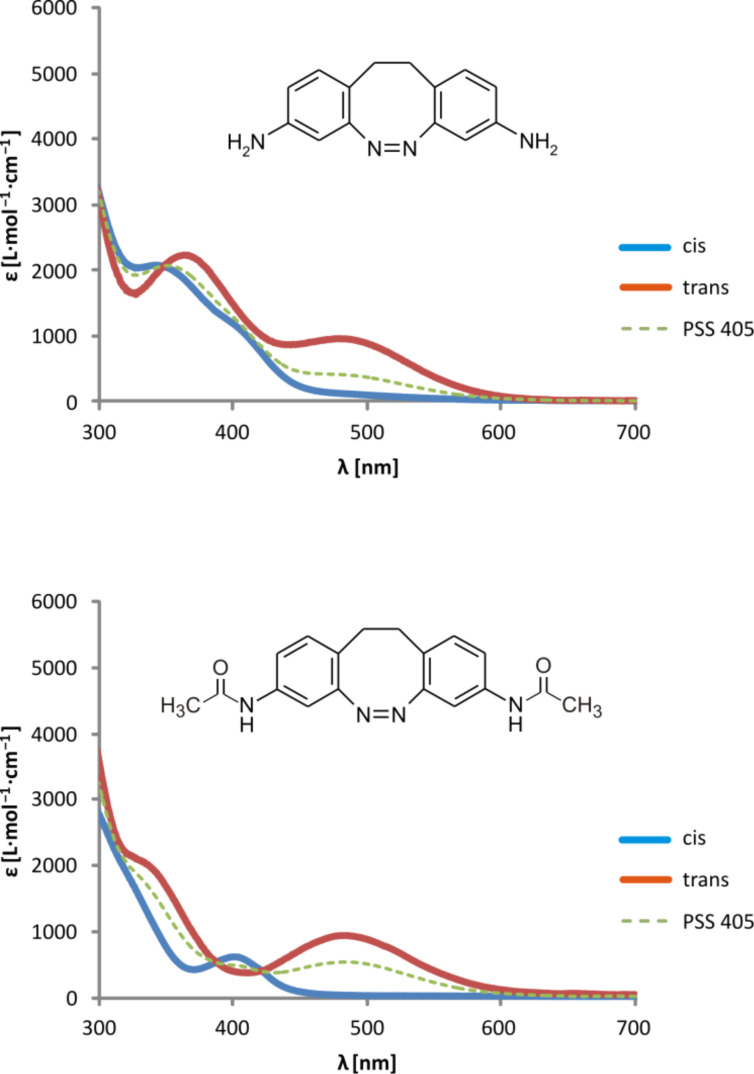
UV–vis spectra of the diazocine derivatives 3,3’-diamino-EBAB **4** and its bisamide derivative **5** in acetonitrile.

**Figure 4:**
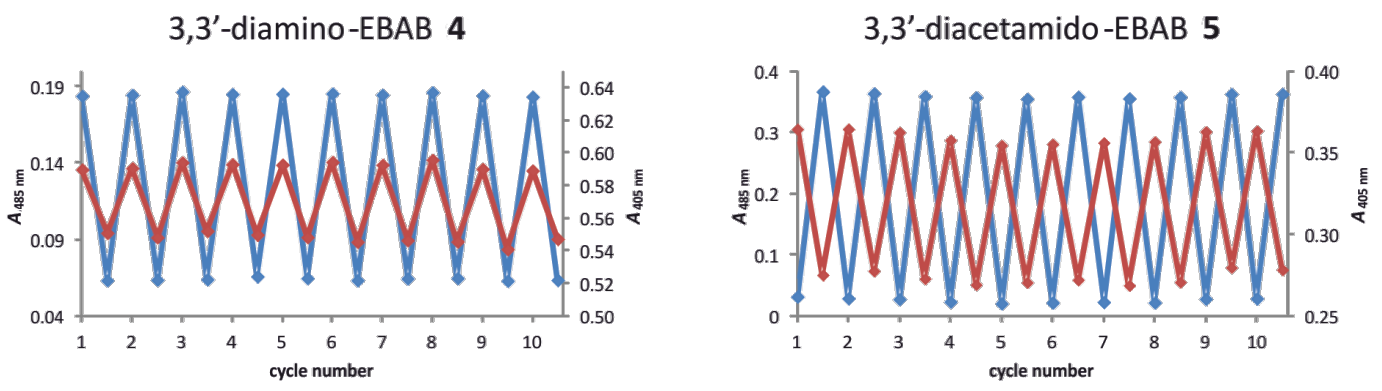
Absorbances of solutions of **4** and **5** in acetonitrile at 405 nm (red) and 485 nm (blue) in the corresponding photostationary states upon alternating irradiation at 405 and 530 nm.

The UV spectrum of *cis*-3,3’-diamino-EBAB **4** exhibits a π–π* transition at 350 nm, and a shoulder at approximately 400 nm, which arises from the n–π* transition of the azobenzene substructure (Figure 3). Irradiation of a solution of **4** in acetonitrile with light of 405 nm leads to isomerization to the *trans* isomer with 34% *trans* form in the photostationary state (determined by ^1^H NMR). The rather low conversion rate is probably due to the overlap of the π–π* and n–π* transitions in the *cis* form. In the *trans* isomer the n–π* transition is shifted to longer wavelengths (~500 nm) and is clearly separated from any other absorption. Irradiation with light at 450–600 nm therefore converts the *trans* isomer completely back to the *cis* form. As compared to the bisamino-substituted EBAB **4** the π–π* and the n–π* bands of the *cis* isomer of the acetamido derivate **5** are better separated (shoulder at 320 nm and 400 nm) (Figure 3). The photostationary state upon irradiation with 405 nm therefore rises to 54% *trans* form. As in the bisamino-substituted system **4**, conversion of **5** back to the *cis* isomer is quantitative within the error limits of ^1^H NMR and UV–vis spectroscopy (Figure 4).

### Thermal stability of the *trans* isomers

Amino and alkylamino substituents in *para* position to the azo group reduce the lifetime of the *cis* states of azobenzenes [15–16]. A dramatically shortened half-life of an *ortho*-dimethylamino-substituted *cis*-phenylazopyridine (*cis*-4-*N*,*N*-dimethylamino(3-phenylazo)pyridine) has also been observed [17]. The reverse effect was observed in our *meta*-substituted *trans*-3,3’-diamino-EBAB **4**. While the unsubstituted *trans*-EBAB **1** exhibits a half-life of 4.5 h in *n*-hexane solution at room temperature, *trans*-3,3’-EBAB **4** isomerizes to the more stable *cis* form with a half-life of 74 h. The corresponding half-life of *trans*-3,3’-acetamido-EBAB **5** is 46 h (Table 1).

**Table 1:** Half-life and photostationary states of EBAB **1** and derivatives.

compound	half life [h]	PSS 405 nm % *trans*	PSS 520 nm % *trans*

EBAB **1**	4.5^a^	92%	<1%
3,3’-diamino-EBAB **4**	74^b^	34%	<1%
3,3’-diacetamido-EBAB **5**	46^b^	54%	<1%
4,4’-diacetamido-EBAB	4.8^c^	70%	<1%

^a^300 K, *n*-hexane [6]; ^b^300 K, MeCN; ^c^293 K, DMSO [9].

### Application as a molecular pincer

In the ^1^H NMR spectra of *cis*-**4** and *cis*-**5** the four protons of the ethylene bridge yield a centred multiplet. This symmetry of the fine structure shows that they divide into two chemically unequal groups of two chemically equal protons. Hence, there is no boat inversion at room temperature on the NMR time scale. According to the X-ray crystal structures the amino nitrogen lone pairs of *cis*-3,3’-diamino EBAB **4** point towards each other and (if extended to more than 6.5 Å) would intersect with an angle of about 65°. If the acetamido groups in **5** are rotated appropriately, the N–H bonds (extended to 10.5 Å) intersect with an angle of about 45°. Thus, the EBAB derivatives **4** and **5** should be suitable as molecular pincers.

**Figure 5:**
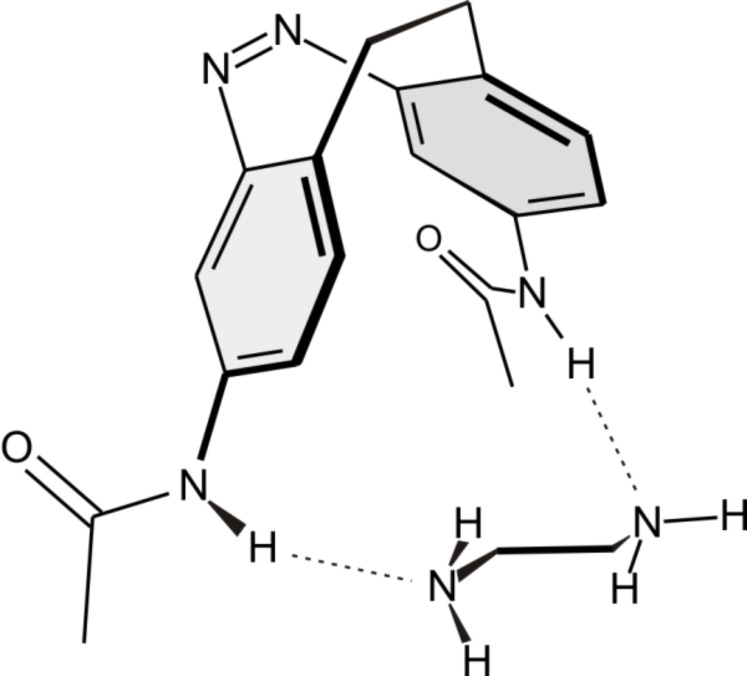
DFT-calculated structure (B3LYP/6-31+G**) of a complex of **5** with ethylenediamine as a conceivable model of the binding mode of 3,3’-diacetamido-EBAB **5**.

To demonstrate this property we studied the binding of ethylenediamine to the two isomers of the acetamido derivative **5** (Figure 5).

We carried out ^1^H NMR titrations of *cis*-**5** as well as of the photostationary mixture of *cis*-**5** and *trans*-**5** upon irradiation with light of a wavelength of 405 nm with ethylenediamine in acetonitrile. The spectra showed a significant chemically induced shift (CIS) of the acetamide protons of both isomers upon addition of ethylenediamine. Equilibrium analysis with respect to the CIS binding isotherms by means of nonlinear least-squares methods (for details see Supporting Information File 1) [18–19] yielded a binding constant for the 1:1 ethylenediamine complex of the *cis* isomer of *K*_a,_*_cis_* = 0.88 ± 0.03 M^−1^. For the corresponding complex of the *trans* isomer a slightly lower binding constant of *K*_a,_*_trans_* = 0.61 ± 0.05 M^−1^ was determined in acetonitrile-*d*_3_ at 25 °C.

## Conclusion

We prepared ethylene-bridged azobenzene (EBAB) derivatives with amino and acetamido substituents in the *meta* position with respect to the azo group (3,3’-diamino-EBAB **4**, and 3,3’-diacetamido-EBAB **5**). In contrast to azobenzene, and in agreement with the parent EBAB, the *cis* isomer is more stable than the *trans* form. Compared to the parent EBAB, which is a very efficient photoswitch, the conversion from the *cis* to the *trans* isomer upon irradiation with 405 nm is reduced to 34% (diamino derivative **4**) and 54% (diacetamido derivative **5**, cf. 92% in the parent compound). The thermal half-life of the *trans* isomer, however, is drastically increased (3,3’-diamino-EBAB **4**: 74 h, 3,3’-diacetamido-EBAB **5**: 46 h). The EBAB derivatives upon photoisomerization perform a pincer-like motion. Diacetamido derivative **5** binds ethylenediamine better in its *cis* (closed) form than in its *trans* configuration (open form).

## Experimental

### General remarks

All chemicals were purchased from commercial sources and used without further purification. All NMR spectra were recorded with instruments of the company Bruker (AC 200, DRX 500, and AV 600). The assignments of the NMR signals were confirmed by the evaluation of COSY, HSQC and HMBC spectra. The chemical shifts of the signals were all referenced to residual solvent peaks. The mass spectra were recorded on a Finnigan MAT 8230 instrument. IR spectra were recorded with a Spectrum 100 instrument from Perkin-Elmer equipped with an ATR unit from the company Loriot-Oriel. Melting points were taken without correction. UV–vis spectra were recorded on a Perkin-Elmer Lambda 14 spectrometer.

### Synthesis

**1,2-Bis(2-nitro-4-aminophenyl)ethane (3):** A solution of 5.0 g 1,2-bis(4-aminophenyl)ethane (24.0 mmol) in 40 mL of sulfuric acid was warmed up to 60 °C and a solution of 4.4 g (52.0 mmol) finely grounded sodium nitrate in 45 mL of sulfuric acid was added dropwise. The mixture was stirred at 60 °C for 6 h and afterwards poured into 200 mL of ice–water. The resulting suspension was neutralised by the addition of an aqueous ammonia solution (32%). The red precipitate was filtered off, washed with water and dried in vacuum over CaCl_2_. Yield: 7.2 g (23.6 mmol, 99%). Mp 247–249 °C; ^1^H NMR (500 MHz, DMSO-*d*_6_) δ 7.06 (d, ^4^*J*_6,2_ = 2.0 Hz, 2H, 6-*H*), 6.99 (d, ^3^*J*_3,2_ = 8.3 Hz, 2H, 3-*H*), 6.78 (dd, ^3^*J*_2,3_ = 8.3 Hz, ^4^*J*_2,6_ = 2.1 Hz, 2H, 2-*H*), 5.58 (s, 4H, 1-N*H*_2_), 2.86 (s, 4H, 7-*H*); ^13^C NMR (126 MHz, DMSO-*d*_6_) δ 149.90 (C_q_, *C*-5), 148.58 (C_q_, *C*-1), 132.85 (d, *C*-3), 121.85 (C_q_, *C*-4), 119.21 (d, *C*-2), 108.48 (d, *C*-6), 33.25 (t, *C*-7); IR (ATR): 3444 (m), 3363 (s), 3234 (w), 3061 (w), 2947 (w), 2877 (w), 1622 (s), 1513 (vs), 1495 (vs), 1324 (vs), 1272 (s), 1263 (s), 829 (s), 818 (s) cm^−1^; EIMS (70 eV) m/z (% relative intensity): 302 (16) [M]^+^, 151 (100); CIMS (isobutane) m/z (% relative intensity): 303 (100) [M + H]^+^.

**(*Z*)-11,12-Dihydrodibenzo[*c*,*g*][1,2]diazocine-3,8-diamine (4):** A suspension of 1,2-bis(2-nitro-4-aminophenyl)ethane (**3**) (1.059 g, 3.5 mmol) in a mixture of 140 mL ethanol and a solution of 8.8 g (220 mmol) sodium hydroxide in 35 mL water was heated to 70 °C. A solution of 6.5 g (36 mmol) glucose in 20 mL water was added, and the reaction mixture was stirred overnight. After cooling to room temperature, 500 mL water was added, and the resulting mixture was extracted three times with 100 mL of ethyl acetate. The organic phase was separated and dried over sodium sulfate, and the solvent was evaporated in vacuum. From the obtained residue the product was isolated by flash chromatography (silica gel, cyclohexane/ethyl acetate 1:1) (254 mg, 1.1 mmol, 30%). Mp 193–196 °C; ^1^H NMR (600 MHz, DMSO-*d*_6_) δ 6.67 (d, ^3^*J*_6,5_ = 8.2 Hz, 2H, 6-*H*), 6.24 (dd, ^3^*J*_5,6_ = 8.2 Hz, ^4^*J*_5,3_ = 2.3 Hz, 2H, 5-*H*), 5.97 (d, ^4^*J*_3,5_ = 2.3 Hz, 2H, 3-*H*), 5.15 (br.s, 4H, 4-N*H*_2_), 2.63 (m_c_, 4H, 7-*H*_a_*,* 7-*H*_b_*,*); ^13^C NMR (150 MHz, DMSO-*d*_6_) δ 155.87 (C_q_, *C*-1), 146.76 (C_q_, *C*-2), 130.15 (d, *C*-6), 115.18 (C_q_, *C*-4), 112.88 (d, *C*-5), 103.11 (d, *C*-3), 30.44 (t, *C*-7); IR (ATR): 3433 (m), 3344 (m), 3433 (m), 2952 (m), 2850 (m), 1703 (w), 1609 (vs), 1570 (m), 1497 (vs), 1455 (s), 1435 (m), 1303 (s), 1272 (s), 1170 (m), 1142 (m), 1094 (m), 1020 (m), 930 (w), 900 (m), 856 (m), 808 (vs) cm^−1^; EIMS (70 eV) m/z (% relative intensity): 238 (100) [M]^+^, 209 (92), 193 (46); CIMS (isobutane) m/z (% relative intensity): 239 (100) [M + H]^+^.

**(*Z*)-N,N'-(11,12-Dihydrodibenzo[*c*,*g*][1,2]diazocine-3,8-diyl)diacetamide (5):** In acetic acid anhydride (25 mL), (*Z*)-11,12-dihydrodibenzo[*c*,*g*][1,2]diazocine-3,8-diamine (**4**) (5 mg, 20 mmol) was dissolved. The solution was stirred at room temperature overnight. Afterwards the solvent was evaporated in vacuum, and the product was obtained as a pale yellow solid (7 mg, 20 mmol, 100%). Mp 220–221 °C; ^1^H NMR (500 MHz, MeCN-*d*_3_) δ 8.25 (s, 2H, 5-N*H*), 7.06 (d, ^4^*J*_3,5_ = 2.2 Hz, 2H, 3-*H*), 7.03 (dd, ^3^*J*_5,6_ = 8.2 Hz, ^4^*J*_5,3_ = 2.2 Hz, 2H, 5-*H*), 6.87 (d, ^3^*J*_3,5_ = 2.2 Hz, 2H, 3-*H*), 2.73 (m, 2H, 7-*H*_a_*,*), 2.70 (m_c_, 4H, 7-*H*_a_*,* 7-*H*_b_), 2.00 (s, 6H, 9-*H*); ^13^C NMR (125 MHz, MeCN-*d*_3_) δ 168.36 (C_q_, *C*-8), 155.27 (C_q_, *C*-1), 137.45 (C_q_, *C*-2), 129.94 (d, *C*-6), 123.17 (C_q_, *C*-4), 117.44 (d, *C*-5), 108.59 (d, *C*-3), 30.29 (t, *C*-7), 22.96 (q, *C*-9); IR (ATR): 3253 (m), 3174 (m), 3102 (m), 3048 (m), 2924 (m), 1711 (m), 1680 (m), 1300 (s), 1260 (s), 1020 (s), 980 (m), 957 (m), 899 (m), 883 (m), 814 (s), 763 (m) cm^−1^. EIMS (70 eV) m/z (% relative intensity): 322 (50) [M]^+^, 252 (92), 209 (100); CIMS (isobutane): m/z (% relative intensity) 323 (100) [M + H]^+^.

## Supporting Information

^1^H NMR spectra of **4** and **5** before and after the irradiation with 405 nm, and ^1^H NMR binding study of 3,3-acetamido-EBAB (**5**) with ethylenediamine. cif-Files of X-ray crystal structures of *cis*-**4** and *cis*-**5**, and gaussian09 input file of the geometry optimization of the complex of *cis*-**5** and ethylenediamine (DFT B3LYP/6-31+G**).

File 1 Additional NMR spectra and ^1^H NMR binding study of 3,3-acetamido-EBAB (**5**) with ethylenediamine.

File 2 Crystallographic information file of compound cis-**4**.

File 3 Crystallographic information file of compound cis-**5**.

File 4 Gaussian09 input file of the geometry optimization of the complex of *cis*-**5** and ethylenediamine.
