# Supplementary material for: Amino-substituted diazocines as pincer-type photochromic switches
Source: Beilstein J Org Chem. 2013 Jan 2;9:1–7. doi: 10.3762/bjoc.9.1 (PMC3566864; doi:10.3762/bjoc.9.1)
Supplement: File 1 — Additional NMR spectra and 1H NMR binding study of 3,3-acetamido-EBAB (5) with ethylenediamine. [file Beilstein_J_Org_Chem-09-01-s001.pdf]

Supporting Information

for

Amino-substituted diazocines as pincer-type photochromic switches

Hanno Sell[1], Christian Näther[2] and Rainer Herges[1]*

Address: [1]Otto-Diels Institut für Organische Chemie, Christian-Albrechts-Universität zu Kiel, Otto-Hahn-Platz 4, 24418 Kiel, Germany and [2]Institut für Anorganische Chemie, Christian-Albrechts-Universität zu Kiel, Max-Eyth-Str. 2, 24418 Kiel, Germany

Email: Rainer Herges* - rherges@oc.uni-kiel.de

* Corresponding author

Additional NMR spectra and ^{1}H NMR binding study of 3,3-acetamido-EBAB (5) with ethylenediamine

^{1}H NMR spectra of **4** and **5** before and after irradiation with light of the wavelength 405 nm

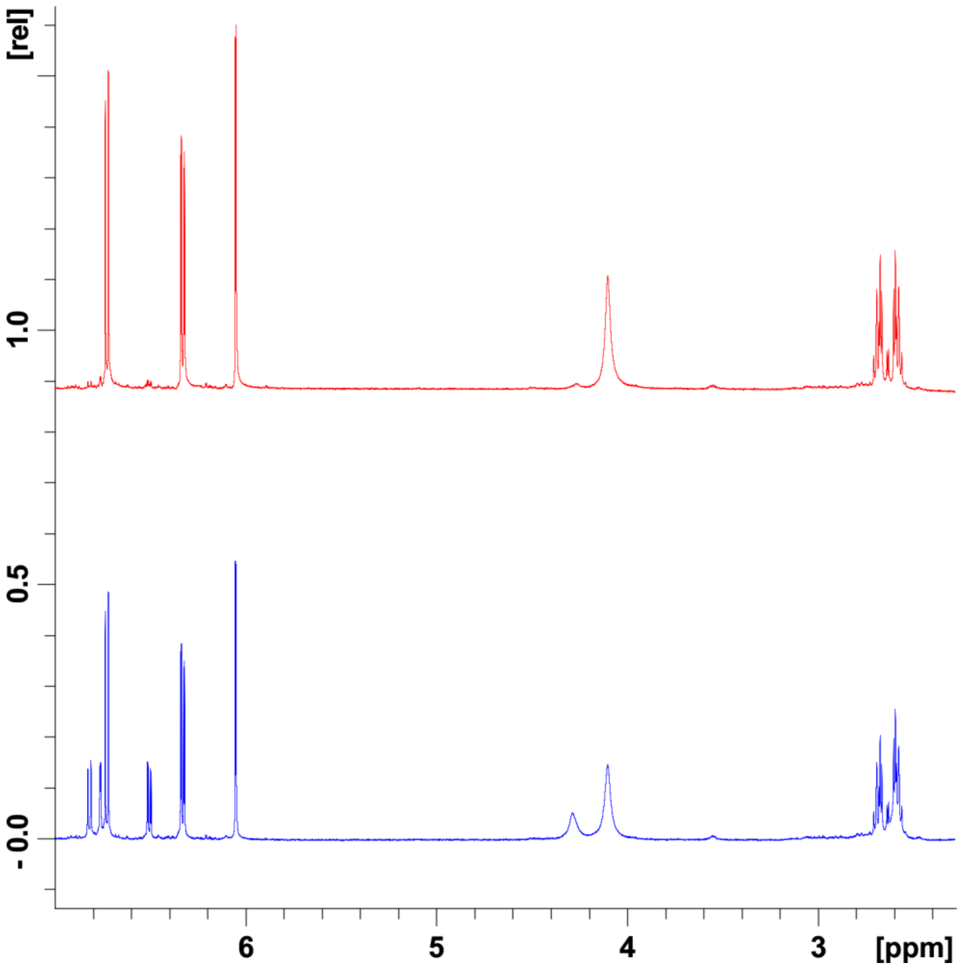

Figure S1: ^{1}H NMR spectra of 3,3'-diamino-EBAB **4** before (red) and after (blue) irradiation with light of the wavelength 405 nm in acetonitrile.

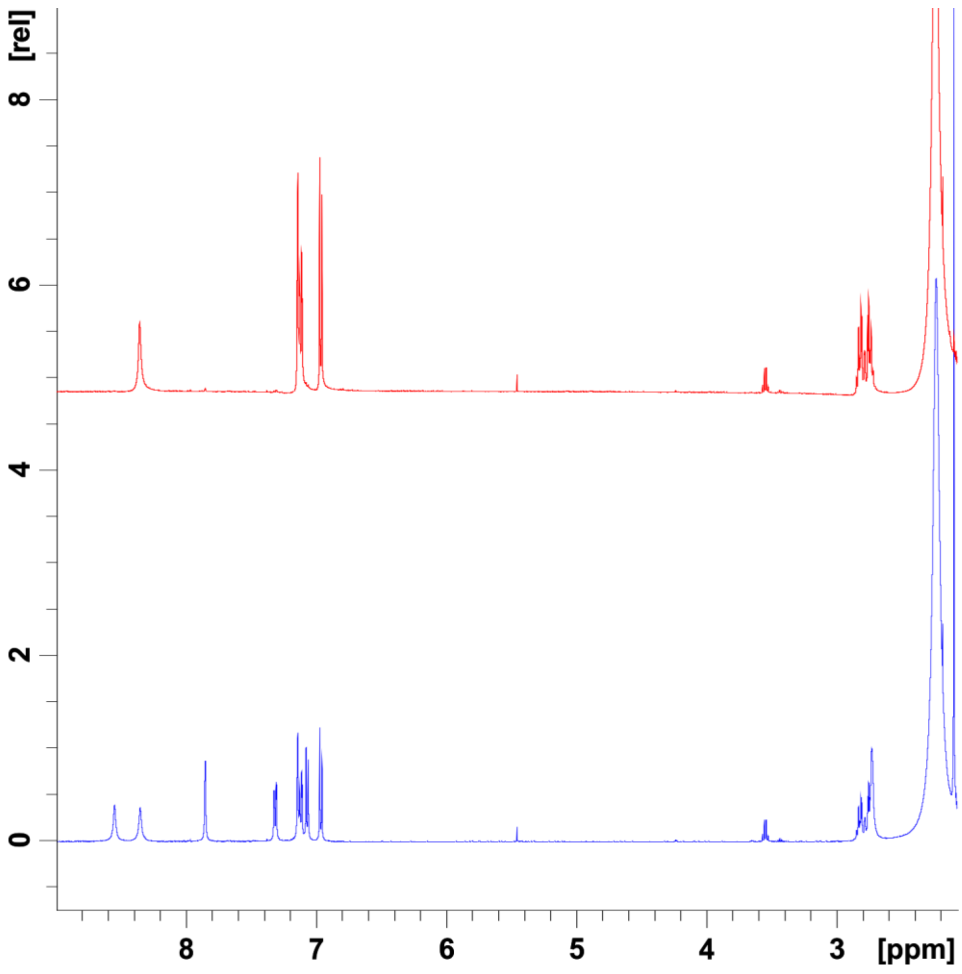

Figuer S2: ^{1}H NMR spectra of 3,3’-diacetamido-EBAB **5** before (red) and after (blue) irradiation with light of the wavelength 405 nm in acetonitrile.

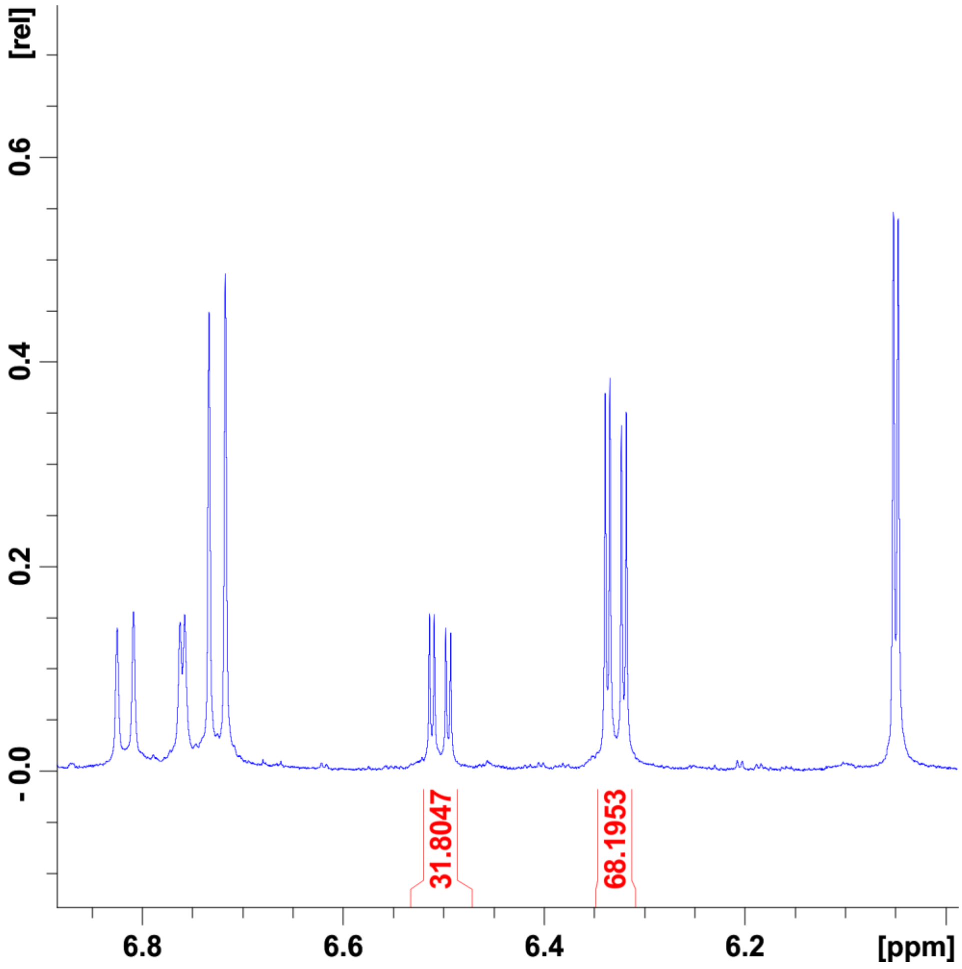

Figure S3: The ^{1}H NMR spectrum of 3,3'-diamino-EBAB **4** in the spectral region of the aromatic protons after irradiation with light of the wavelength 405 nm. The *cis*-**4** to *trans*-**4** ratio was determined by the integration of the ^{1}H NMR signals of the phenyl proton in *para* position to the azo group in both isomers.

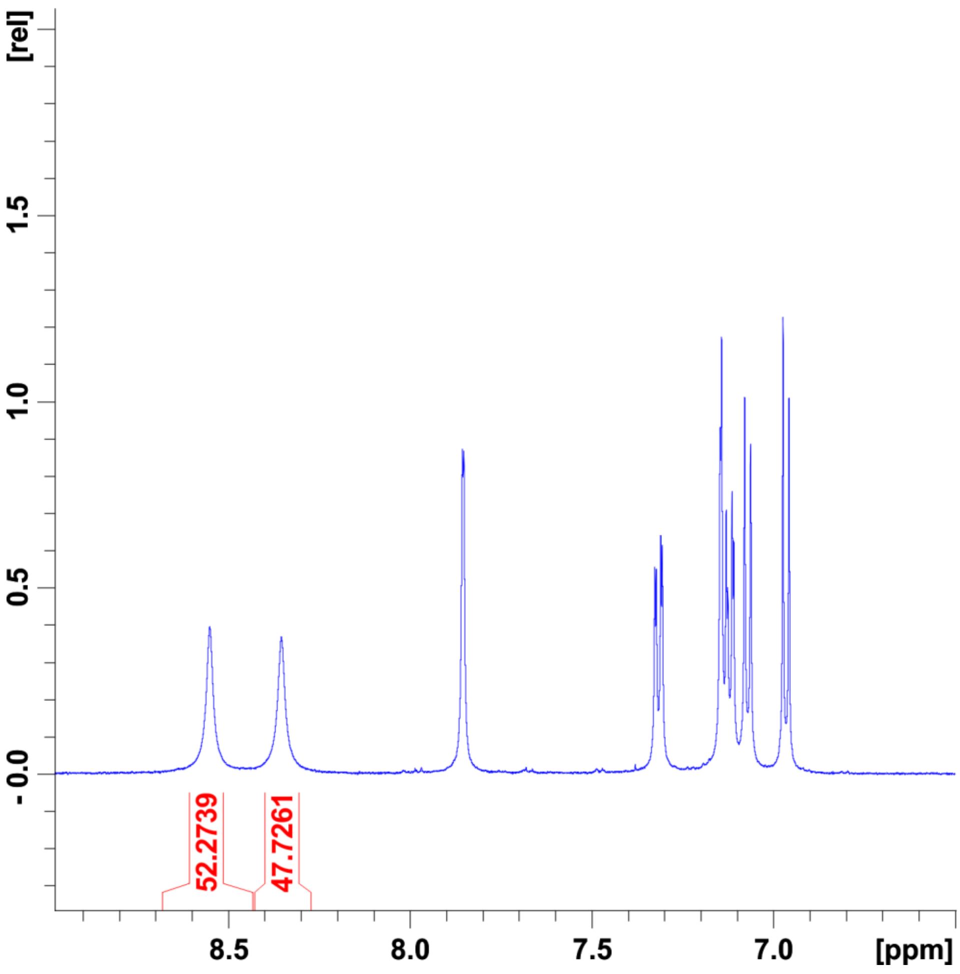

Figure S4: The ^{1}H NMR spectrum of 3,3'-diacetamido-EBAB **5** in the spectral region of the aromatic protons after irradiation with light of the wavelength 405 nm. The *cis*-**5** to *trans*-**5** ratio was determined by the integration of the ^{1}H NMR signals of the protons of the amide group in both isomers.

^{1}H NMR binding study of 3,3'-acetamido-EBAB **5** with ethylenediamine

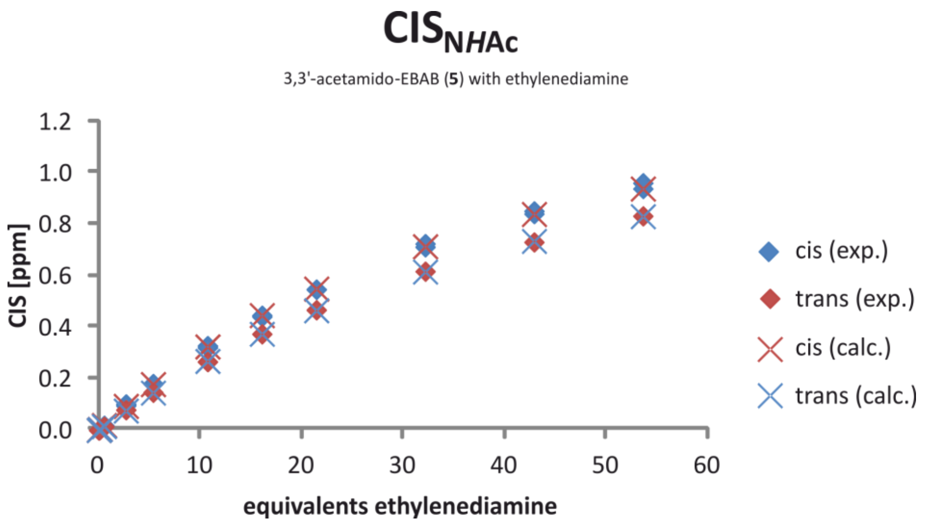

Figure S5: Graphic representation of the results of the ^{1}H NMR titration of 3,3'-acetamido-EBAB **5** with ethylenediamine in MeCN.

Stock solutions used for the titration:

stock solution of **5**: 15.85 mg 3,3-acetamido-EBAB **5** in 1.0 mL MeCN-d_3

ethylenediamine stock solution I: 2.49 mg ethylenediamine in 500 µL MeCN-d_3

ethylenediamine stock solution II: 101.40 mg ethylenediamine in 1.0 mL MeCN-d_3

Table S1: Composition of the samples of the ^{1}H NMR titration of 3,3'-acetamido-EBAB **5** with ethylenediamine.

Nr.	volume of MeCN-d_3 [µL]	volume of stock solution of **5** [µL]	volume of ethylenediamine stock solution I [µL]	volume of ethylenediamine stock solution II [µL]	Irradiation state
1	340	160	0	0	PSS 530
2	340	160	0	0	PSS 405
3	340	160	10	0	PSS 530
4	340	160	10	0	PSS 405
5	340	160	50	0	PSS 530
6	340	160	50	0	PSS 405
7	340	160	50	10	PSS 530
8	340	160	50	10	PSS 405
9	340	160	50	22.5	PSS 530
10	340	160	50	22.5	PSS 405
11	340	160	50	47.5	PSS 530
12	340	160	50	47.5	PSS 405
13	340	160	50	72.5	PSS 530
14	340	160	50	72.5	PSS 405
15	340	160	50	97.5	PSS 530
16	340	160	50	97.5	PSS 405
17	340	160	50	122.5	PSS 530
18	340	160	50	122.5	PSS 405
19	340	160	50	147.5	PSS 530
20	340	160	50	147.5	PSS 405
21	340	160	50	172.5	PSS 530
22	340	160	50	172.5	PSS 405

Analysis of the fine structure of the ^{1}H NMR signals of the protons of the ethylene bridge of *cis*-3,3-diamino-EBAB (*cis*-**4**)

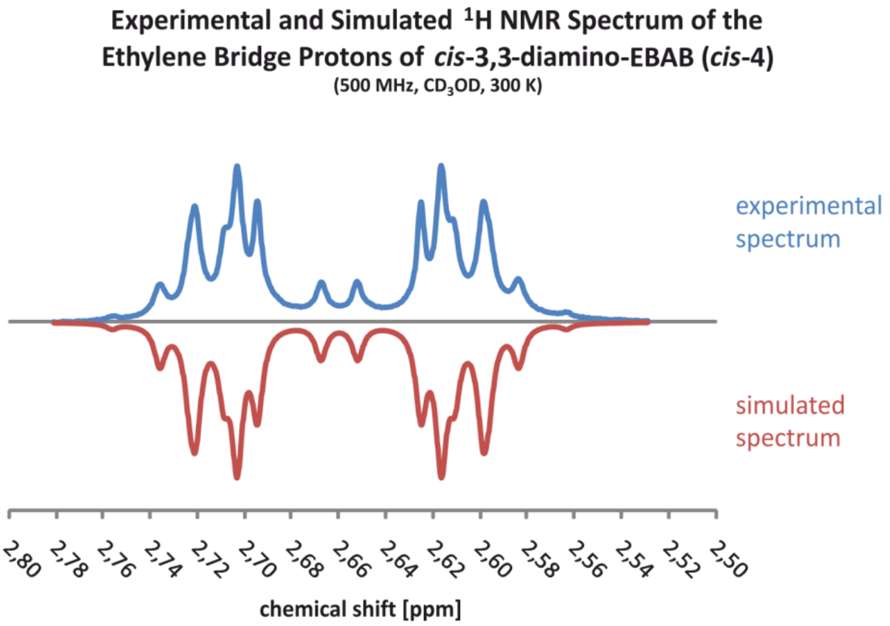

Figure S6: Experimental and simulated ^{1}H NMR spectrum of the protons of the ethylene bridge of *cis*-3,3-diamino-EBAB (*cis*-**4**)

Table S2: Parameters of the ^{1}H NMR signals of the protons of the ethylene bridge of *cis*-3,3-diamino-EBAB (*cis*-**4**) in CD_3OD at 300 K.

parameter	value
ν (7-H_a)	2.667 ppm
ν (7-H_b)	2.652 ppm
$^{2}J_{a,a'}$	9.6 Hz
$^{2}J_{b,b'}$	9.6 Hz
$^{3}J_{a,b}$	−14.7 Hz
$^{3}J_{a,b'}$	6.0 Hz
